# Comparative Evidence for Intrahepatic Cholestasis of Pregnancy Treatment With Traditional Chinese Medicine Therapy: A Network Meta-Analysis

**DOI:** 10.3389/fphar.2021.774884

**Published:** 2021-11-30

**Authors:** Yinxiao Jiang, Haotian Li, Dan Song, Penghui Ye, Nuo Xu, Yuan Chen, Wenwen Zhang, Qichao Hu, Xiao Ma, Jianxia Wen, Yeyu Li, Yanling Zhao

**Affiliations:** ^1^ State Key Laboratory of Southwestern Chinese Medicine Resources, School of Pharmacy, Chengdu University of Traditional Chinese Medicine, Chengdu, China; ^2^ Department of Pharmacy, The Fifth Medical Center of PLA General Hospital, Beijing, China; ^3^ School of Food and Bioengineering, Xihua University, Chengdu, China; ^4^ Hospital of Chengdu University of Traditional Chinese Medicine, School of Clinical Medicine, Chengdu University of Traditional Chinese Medicine, Chengdu, China

**Keywords:** intrahepatic cholestasis of pregnancy, ursodeoxycholic acid, Yinchenhao decoction, traditional Chinese medicine, network meta-analysis

## Abstract

**Background:** Intrahepatic cholestasis of pregnancy (ICP) seriously threatens the health of pregnant women and newborns. A various number of Chinese prescriptions and patent medicines combined with ursodeoxycholic acid (UDCA) are used for treating ICP in China. However, there are still many doubts in choosing the suitable therapeutic drugs for the treatment of ICP in clinical practice.

**Methods:** Several electronic databases, including PubMed, Embase, Cochrane Library, China National Knowledge Infrastructure (CNKI), China Biology Medicine disc (CBM), Wanfang, and VIP, were comprehensively searched from the database inception to February 22, 2021. Randomized controlled trials (RCTs) reporting the use of UDCA only, Chinese prescriptions plus UDCA, and patent medicine plus UDCA for the treatment of ICP were collected according to their inclusion and exclusion criteria. Cochrane Reviewers’ Handbook version 5.2 was applied for the risk assessment of the included trials. STATA 16.0 software was used for network meta-analysis (NMA). The pruritus score and the serum levels of total bile acid (TBA), alanine aminotransferase (ALT), and aspartate transaminase (AST) in ICP patients served as the primary outcomes. Moreover, this study had been registered in PROSPERO (https://www.crd.york.ac.uk/PROSPERO/#joinuppage), and the registration number is CRD42020188831.

**Results:** Thirty-eight RCTs comprising 3,841 patients meeting the inclusion criteria were included in the network meta-analysis. The NMA results showed that compared with UDCA used alone, Yinchenhao decoction (seven different Chinese prescriptions or patent medicines) plus UDCA dramatically alleviated the primary outcomes of ICP, including the pruritus score, as well as the serum levels of TBA, ALT, and AST. The NMA results showed that the optimal drug ratio for the treatment of ICP was different from the dosage ratio of traditional Yinchenhao decoction. Significantly, the intervention plan f (IP-f) group [the similar prescription of Yinchenhao decoction 2 (*Artemisia capillaris Thunb* >15 g, *Gardenia* >9 g, and *Rhubarb* <5 g) + UDCA] was the best therapeutics among the eight therapies.

**Conclusion:** Overall, the combined use of Chinese prescriptions or patent medicine with UDCA was generally better than UDCA used alone. The dose of IP-f might be a beneficial therapeutic method for the clinical medication of ICP.

**Clinical Trail Registration:** https://www.crd.york.ac.uk/, identifier CRD42020188831.

## Introduction

Intrahepatic cholestasis of pregnancy (ICP) is the most common hepatic disease in pregnant women, with high cases in different regions all over the world (Piechota and Jelski, 2020). In general, ICP often occurs in the middle and later stages of pregnancy and rarely occurs in early pregnancy (Wongjarupong et al., 2020). The primary clinical symptoms of ICP include maternal pruritus and the serum level of total bile acid (TBA) more than 10 μmol/l (Walker et al., 2002; Glantz et al., 2004). Simultaneously, a study found that the alcohol dehydrogenase activity was increased in women with ICP compared with healthy pregnant and non-pregnant women. Alcohol dehydrogenase activity could become a new outcome that diagnoses ICP in the future (Jelski et al., 2020). In addition, in-depth research on ICP found that genetic defects in canalicular transporters were related to ICP. Moreover, ICP is a major threat to the health and life of pregnant women and newborns. For pregnant women, the adverse perinatal outcomes caused by ICP are mainly increased postpartum bleeding (Hou, 1988; Arthuis et al., 2020). Simultaneously, a long-term investigation found that women with a history of ICP had a higher risk of suffering the following diseases: cholelithiasis, cholecystitis, hypothyroidism, and diseases of the pancreas and the digestive system (Hämäläinen et al., 2019). For newborns, the threats caused by ICP are mainly as follows: premature birth, lesser weight, amniotic fluid contamination, cardiac arrhythmias, infant respiratory distress syndrome, and the most serious is sudden fetal death (Arthuis et al., 2020; Brouwers et al., 2015; Lin et al., 2019).

Currently, ursodeoxycholic acid (UDCA) is the only first-line drug approved by the U.S. Food and Drug Administration for the treatment of cholestasis. According to clinical trials, the therapeutic effectiveness and safety of UDCA have been fully proven for relieving symptoms in patients with ICP in the past several decades (Wood et al., 2018). However, UDCA is not effective in treating part diseases related to cholestasis and is also limited to a certain extent (Shah and Kowdley, 2020). As one of the most significant challenges in drug discovery, more effective and reasonable ICP intervention drugs still need to be further developed. At present, the combination use of UDAC with other drugs is often applied for treating ICP in clinical practice with good curative effect and few adverse reactions. In China, traditional Chinese medicine prescriptions and Chinese patent medicines combined with UDCA are widely applied for the treatment of ICP. However, these therapies are different from each other and lack systematic research. Furthermore, compatible dosage of traditional Chinese medicine is particularly important in clinical practice, and the effective dose is difficult to determine. As different prescriptions have different medicinal properties with complex systems, it is difficult to systematically study prescriptions for the treatment of ICP. Simultaneously, among these prescriptions, we found that the famous Yinchenhao decoction is the most commonly used prescription.

Traditional Chinese medicine Yinchenhao decoction consist of *Artemisia capillaris Thunb*, *Gardenia*, and *Rhubarb*. Among these medicinal materials, *A. capillaris Thunb* is the most important medicinal material. *A. capillaris Thunb* is also named Yinchenhao in China. Its medicinal part is the dry above-ground part. It has multiple chemical components such as coumarin compounds (e.g., 6,7-dimethylesculetin and sabandin A), flavonoids (e.g., luteolin and arcapillin), and organic acids (e.g., rhein and ferulic acid, 2-ethyl furan, and irisone) (Huang et al., 2021). Therefore, it has multiple pharmacological activities including anti-inflammatory, anti-liver injury, anti-liver fibrosis, and anti-tumor (Liu and Zhao, 2017; Gao et al., 2016; Kim et al., 2018; Nam et al., 2018; Tsui et al., 2018; Yan et al., 2018; Jung et al., 2018). For example, a study found that the essential oil of *A. capillaris Thunb* could improve the level of alanine aminotransferase (ALT) and aspartate transaminase (AST) on CCl_4_-induced liver injury in mice. In addition, scoparone is an active ingredient of *A. capillaris Thunb* (Gao et al., 2016).

Based on the previous clinical studies, a network meta-analysis of Chinese prescriptions or patent medicines combined with UDCA in patients with ICP was comprehensively performed. This study was aimed to assess the effectiveness of different treatment methods and the effective dose of these prescriptions based on Yinchenhao decoction so that the clinical efficacy of Yinchenhao decoction can be approved by the international community and possibly enter the international market.

## Materials and Methods

The methodology of this study was in accordance with the network meta-analysis criteria of PRISMA (Moher et al., 2009) and the PRISMA Extension Statement (Hutton et al., 2015). This study has been registered in PROSPERO (https://www.crd.york.ac.uk/PROSPERO/#joinuppage), and the registration number is CRD42020188831.

### Ethical Approval and Consent to Participate

As this study does not involve animal and patient experiments, the ethical approval and consent to participate are not applicable.

### Search Strategy

PubMed, Embase, Cochrane Library, China National Knowledge Infrastructure (CNKI), China Biology Medicine disc (CBM), Wanfang database, and VIP database were comprehensively searched from database inception to February 22, 2021. Intrahepatic cholestasis of pregnancy, UDCA, *Artemisia capillaris Thunb*, Yinzhihuang, salvia, and Chinese medicine were searched as keywords. All databases were retrieved twice. For the first time, the keyword composition including intrahepatic cholestasis of pregnancy, ursodeoxycholic acid, and interventions (*Artemisia capillaris Thunb* or Yinzhihuang or salvia or Chinese medicine) were used to retrieve key information from the databases. For the second time, we used the keyword composition including intrahepatic cholestasis of pregnancy and ursodeoxycholic acid to check if we missed part of the studies. The search strategy in PubMed is listed in Table 1. The search strategy in other databases is similar to the search strategy in PubMed.

**TABLE 1:** Search strategy in PubMed.

Search number	Query	Sort by	Filters	Results
1	{[Intrahepatic cholestasis of pregnancy (MeSH Terms)] AND [ursodeoxycholic acid (MeSH Terms)]} AND (*Artemisia capillaris Thunb* OR yinzhihuang OR salvia OR Chinese medicine)	Most recent	Randomized controlled trial	0
2	[Intrahepatic cholestasis of pregnancy (MeSH Terms)] AND [ursodeoxycholic acid (MeSH Terms)]	Most recent	Randomized controlled trial	16
	Search Details			
1	{[“cholestasis, intrahepatic” (MeSH Terms)] OR [“cholestasis” (All Fields) AND “intrahepatic” (All Fields)] OR “intrahepatic cholestasis” (All Fields) OR [“intrahepatic” (All Fields) AND “cholestasis” (All Fields)]} AND “pregnancy” (MeSH Terms) AND “ursodeoxycholic acid” (MeSH Terms) AND {[“artemisia” (MeSH Terms) OR “artemisia” (All Fields) OR [“artemisia” (All Fields) AND “capillaris” (All Fields)] OR “artemisia capillaris” (All Fields)] AND “Thunb” (All Fields) OR [“yin zhi huang” (Supplementary Concept) OR “yin zhi huang” (All Fields) OR “Yinzhihuang” (All Fields)] OR [“salvia” (MeSH Terms) OR “salvia” (All Fields) OR “salvias” (All Fields) OR “salviae” (All Fields)] OR [“chin med” (Journal) OR [“Chinese” (All Fields) AND “medicine” (All Fields)] OR “Chinese medicine” (All Fields)]} AND [randomized controlled trial (Filter)]			
2	{[“cholestasis, intrahepatic” (MeSH Terms) OR [“cholestasis” (All Fields) AND “intrahepatic” (All Fields)] OR “intrahepatic cholestasis” (All Fields) OR [“intrahepatic” (All Fields) AND “cholestasis” (All Fields)]} AND “pregnancy” (MeSH Terms) AND “ursodeoxycholic acid” (MeSH Terms)] AND [randomized controlled trial (Filter)]			

### Selection Criteria

Two investigators independently examined the titles and abstracts of all searched databases to access the trials for inclusion. The inclusion criteria were as follows: randomized controlled trial (RCT); the patient has itchy skin as the main symptom during pregnancy; and the level of related indicators including TBA, ALT, and AST increased. Exclusion criteria were as follows: other liver and gallbladder diseases such as viral hepatitis. Document criteria were as follows: treatment interventions including UDCA, UDCA plus Yinzhihuang oral liquid, UDCA plus salvia injection, and UDCA plus traditional Chinese medicine prescription were included.

### Basis for Grouping

In this study, we found that most of the traditional Chinese medicine prescriptions used to treat ICP are associated with Yinchenhao decoction. Traditionally, Yinchenhao decoction consists of *A. capillaris Thunb*, *Gardenia*, and *Rhubarb*. Therefore, the included traditional Chinese medicine prescriptions in this study were divided into five groups based on the different doses of Yinchen, Zhizi, and Dahuang. Finally, all studies were divided into seven groups based on the treatment intervention plans as follows: intervention plan a (IP-a): UDCA; intervention plan b (IP-b): Yinzhihuang oral liquid + UDCA; intervention plan c (IP-c): salvia injection + UDCA; intervention plan d (IP-d): Yinchenhao decoction (*A. capillaris Thunb* >15 g, *Gardenia* >9 g, and *Rhubarb* >5 g) + UDCA; intervention plan e (IP-e): the similar prescription of Yinchenhao decoction 1 (*A. capillaris Thunb* >15 g, *Gardenia* <9 g, and *Rhubarb* >5 g) + UDCA; intervention plan f (IP-f): the similar prescription of Yinchenhao decoction 2 (*A. capillaris Thunb* >15 g, *Gardenia* >9 g, and *Rhubarb* <5 g) + UDCA; intervention plan g (IP-g): the similar prescription of Yinchenhao decoction 3 (*A. capillaris Thunb* >15 g, *Gardenia* <9 g, and *Rhubarb* <5 g) + UDCA; and intervention plan h (IP-h): other traditional Chinese medicine prescriptions (*A. capillaris Thunb* <15 g, *Gardenia* <9 g, and *Rhubarb* <5 g) + UDCA. Among these groups, IP-a was the control group (Table 2). The details of the botanical drugs are listed in Supplementary Table S1.

**TABLE 2:** Basis for grouping.

Intervention plan	Acronym	Group standard/interventions
a	IP-a or a	UDCA
b	IP-b or b	Yinzhihuang oral liquid + UDCA
c	IP-c or c	Salvia injection + UDCA
d	IP-d or d	Yinchenhao decoction (*Artemisia capillaris Thunb* >15 g, *Gardenia* >9 g, and *Rhubarb* >5 g) + UDCA
e	IP-e or e	The similar prescription of Yinchenhao decoction 1 (*Artemisia capillaris Thunb* >15 g, *Gardenia* <9 g, and *Rhubarb* >5 g) + UDCA
f	IP-f or f	The similar prescription of Yinchenhao decoction 2 (*Artemisia capillaris Thunb* >15 g, *Gardenia* >9 g, and *Rhubarb* <5 g) + UDCA
g	IP-g or g	The similar prescription of Yinchenhao decoction 3 (*Artemisia capillaris Thunb* >15 g, *Gardenia* <9 g, and *Rhubarb* <5 g) + UDCA
h	IP-h or h	Other traditional Chinese medicine prescriptions (*Artemisia capillaris Thunb* <15 g, *Gardenia* <9 g, and *Rhubarb* <5 g) + UDCA

### Outcomes

As the therapeutic effects of Yinchenhao decoction on ICP is the main question, the pruritus score and the levels of ALT, AST, and TBA were set as the primary outcomes. Among these indices, pruritus score complied with the standards that Ribalta et al. (1991) set (0 marks: no pruritus; one point: occasional pruritus; two points: intermittent pruritus and symptoms are not fluctuating; three points: intermittent pruritus and symptoms are fluctuating; and four points: continuous pruritus at day and night).

### Data Extraction and Quality Assessment

Excel 2010 was used to collect the basic characteristics of patients or participants such as intervention, comparisons, and outcomes by two investigators. The basic characteristics of patients, including intervention plan, number of patients, age, pregnancy time, and period of treatment, were collected (Table 3). Primary outcomes included pruritus score and the level of ALT, AST, and TBA. Simultaneously, the missing or repeated data were excluded. Quality assessment was performed using Review Manager 5.3 according to the Cochrane Handbook for Systematic Reviews of Interventions, version 5.2. Disagreements on the assessment of data were resolved by discussion, and consensus was reached in all cases.

**TABLE 3:** Basic characteristics of randomized controlled trials included in network meta-analysis.

No	Study	Intervention		Number of patients	Ages (*x̄* ± *s*)	Pregnancy time/week (*x̄* ± *s*)	Period of treatment (days)	Outcomes
1	Jiang (2014)	ab	b	30	26.7 ± 2.8	33.19 ± 0.97	20	1234
			a	30	27.0 ± 2.6	33.16 ± 0.77	20	
2	Yuan and Lan (2014)		b	42	29.50 ± 5.10	34.5 ± 6.70	10	1234
			a	42	29.80 ± 5.70	33.90 ± 7.20	10	
3	Deng (2015)		b	30	–	–	10	13
			a	30	–	–	10	
4	Dai (2016)		b	160	26.8 ± 2.3	33.1 ± 0.96	20	2
			a	160	26.7 ± 2.7	33.23 ± 0.93	20	
5	Wu and Yang (2016)		b	43	26.81 ± 4.69	33.52 ± 1.03	20	234
			a	43	26.92 ± 4.77	33.48 ± 1.11	20	
6	Bo (2017)		b	43	24.2 ± 4.8	30.2 ± 3.4	10	234
			a	43	24.9 ± 4.5	30.8 ± 3.1	10	
7	Zhang (2017)		b	62	29.7 ± 3.8	–	14	234
			a	62	28.6 ± 4.5	–	14	
8	Liang (2018)		b	51	20.86 ± 3.35	31.19 ± 2.31	10	234
			a	51	28.77 ± 4.41	31.23 ± 2.26	10	
9	Yu and Chen (2018)		b	33	22.4 ± 4.3	24.4 ± 2.1	14	234
			a	33	23.1 ± 4.6	25.6 ± 3.6	14	
10	Fang et al. (2009)	ac	c	72	27.3 ± 2.6	33.8 ± 2.1	14	134
			a	56	25.8 ± 3.1	34.1 ± 2.3	14	
11	Pan et al. (2013)		c	45	–	–	7	1234
			c	35	–	–	7	
12	Peng (2015)		c	43	26.1 ± 1.4	35.8 ± 0.9	7	1234
			a	42	25.6 ± 1.2	35.6 ± 0.7	7	
13	Hua (2010)	ad	d	32	–	>28	14	23
			a	28	–	>28	14	
14	Wu (2010)		d	30	–	–	10	234
			a	25	–	–	10	
15	Liu and Yang (2014)		d	71	28.31 ± 5.28	26.93 ± 5.14	14	234
			a	71	27.02 ± 5.14	27.26 ± 3.49	14	
16	Fu (2017)		d	40	28.6 ± 2.5	29.1 ± 2.2	14	234
			a	40	28.4 ± 2.9	29.8 ± 1.9	14	
17	Luo et al. (2019)		d	44	34.88 ± 6.37	29.51 ± 3.09	14	234
			a	44	35.26 ± 6.43	29.67 ± 3.18	14	
18	Zhu and Huang (2008)	ae	e	35	25.1 ± 2.8	34.5 ± 1.9	21	13
			a	25	23.9 ± 2.3	35.1 ± 2.2	21	
19	Wang and Lai (2011)		e	35	24.3 ± 4.5	37 ± 2.14	10	3
			a	35	25.3 ± 3.5	36.43 ± 2.57	10	
20	Wang et al. (2016)		e	55	25.1 ± 4.2	>32	14	123
			a	55	25.3 ± 3.8	>32	14	
21	Wang and Hu (2007)	af	f	36	–	>28	14	234
			a	31	–	>28	14	
22	Xie et al. (2015)		f	36	27.06 ± 4.93	34.4 ± 2.6	21	1234
			a	32	26.87 ± 5.07	35.0 ± 2.4	21	
23	Qiu et al. (2017)		f	46	29.63 ± 3.22	31.78 ± 3.67	7	1234
			a	46	30.11 ± 3.3	32.41 ± 3.52	7	
24	Fang et al. (2018)		f	40	28.7 ± 7.1	32.4 ± 9.2	14	234
			a	40	28.5 ± 6.9	32.9 ± 9.7	14	
25	Guo and Pei (2018)		f	19	38 ± 2.12	30.10 ± 2.42	21	1234
			a	19	38 ± 2.02	30.02 ± 2.52	21	
26	Wang (2009)	ag	g	72	–	>28	7-14	134
			a	62	–	>28	7-14	
27	Wang and Du (2011)		g	90	–	–	10	3
			a	38	–	–	10	
28	Lv (2013)		g	35	26.3 ± 2.4	35.7 ± 1.8	12	23
			a	24	25.03 ± 2.9	35.4 ± 1.4	12	
29	Han et al. (2013b)		g	124	–	–	14	1234
			a	118	–	–	14	
30	Chen et al. (2014)		g	62	27.4 ± 3.1	>28	14	1234
			a	46	27.4 ± 3.1	>28	14	
31	He et al. (2014)		g	29	27.8 ± 3.4	–	20	234
			a	32	27.8 ± 3.4	–	20	
32	Yin and Zhou (2015)		g	30	–	30–34	14	1234
			a	30	–	30–34	14	
33	Gao and Zhao (2017)		g	75	27.42 ± 2.47	32.19 ± 3.37	14	12
			a	75	27.14 ± 2.42	32.24 ± 3.85	14	
34	Luo and Chen (2017)		g	43	25.63 ± 4.43	–	14	2
			a	43	26.50 ± 2.41	–	14	
35	Hu and Yang (2013)	ah	h	88	27.5 ± 4.6	31.2 ± 5.7	20	234
			a	80	27.9 ± 4.3	31.3 ± 5.5	20	
36	Wei (2016)		h	30	28.5 ± 6.1	32.3 ± 3.6	14	234
			a	30	30.5 ± 5.2	31.5 ± 4.3	14	
37	Nie and Lu (2019)		h	40	26.78 ± 2.45	32.24 ± 3.85	14	1234
			a	40	25.68 ± 2.21	32.24 ± 3.85	14	
38	Gu et al. (2014)	adh	d	36	27.8 ± 6.2	31.5 ± 2.8	20	234
			a	47	28.2 ± 5	31.1 ± 3.1	20	
			h	54	29.2 ± 4.9	32.4 ± 2.4	20	

Notes: a: UDCA, b: Yinzhihuang oral liquid + UDCA, c: salvia injection + UDCA; d: Yinchenhao decoction + UDCA, e: the similar prescription of Yinchenhao decoction 1 + UDCA, f: the similar prescription of Yinchenhao decoction 2 + UDCA, g: the similar prescription of Yinchenhao decoction 3 + UDCA, and h: other traditional Chinese medicine prescriptions + UDCA, 1: pruritus score, 2: TBA, 3: ALT, and 4: AST.

### Statistical Analysis

The random-effects model with STATA (version 16.0) was selected to perform the network meta-analysis. All outcomes were continuous outcomes. Therefore, a standardized mean difference (SMD) was selected to calculate the effect size with 95% CI. The intervention plan a (IP-a) is the public control group. The study that does not have IP-a was filled with IP-a data according to the following method. The “mean” of this study is the average of all means of IP-a. The “SD” is more than double the maximum SD of IP-a. The “*n*” is zero. Global inconsistency between direct and indirect sources of evidence was assessed by fitting the inconsistency model. When inconsistency is not significant (*p* > 0.05), it was assessed further to fit the consistency model. We further estimated the ranking probabilities of all treatments that were at different possible ranks for all intervention. At the same time, the result of treatment sequence was summarized and showed with the help of surface under the cumulative ranking curve (SUCRA). Finally, according to the result of SUCRA value, we selected two main outcomes to synthetically analyze the treatment effect of each intervention.

## Results

### Basic Characteristics of Included Randomized Controlled Trials

**FIGURE 1:**
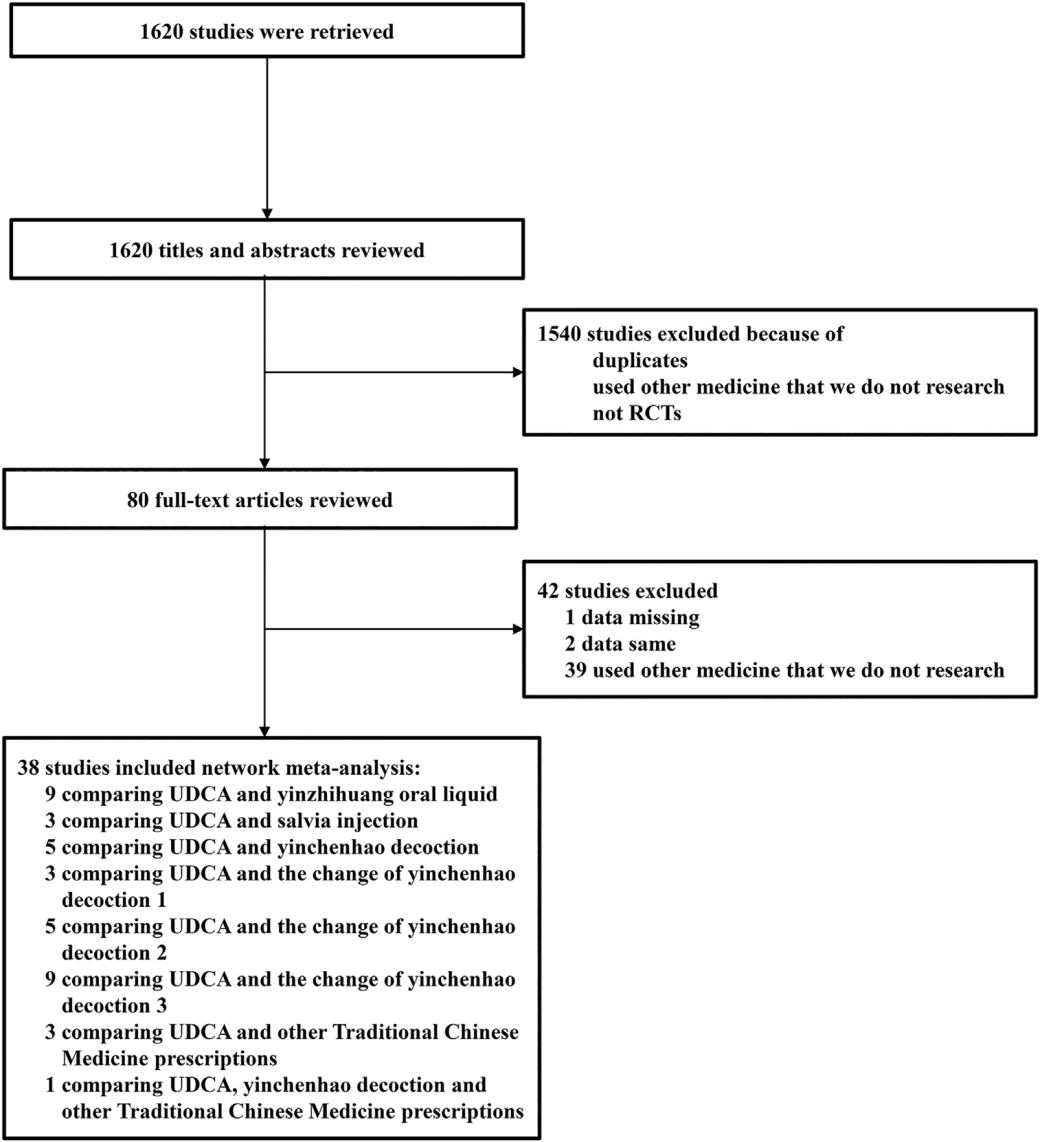
Flow chart of study selection.

A total of 1,620 studies were retrieved from database searches as potentially eligible records. Among them, 1,540 studies were excluded based on reviewed titles and abstracts. Eighty studies were reviewed for further assessment. For the preserved records, 42 studies were excluded due to missing data, same data, or use of other medicine. Finally, 38 RCTs with 3,841 patients with ICP who met the criteria were included in the network meta-analysis. The flow chart of study selection is shown in Figure 1. The basic characteristics of randomized controlled trials included in network meta-analysis is shown in Table 3. The number of patients in each study trial ranged from 32 to 242. Among the included trials, 1,783 patients were included in IP-a; 494 patients were included in IP-b; 160 patients were included in IP-c; 253 patients were included in IP-d; 125 patients were included in IP-e; 177 patients were included in IP-f; 560 patients were included in IP-g; and 212 patients were included in IP-h. The age of the participants ranged from 20.86 to 38 years. The mean pregnancy time of patients ranged from 22.4 to 38 weeks. The period of treatment ranged from 7 to 21 days. Furthermore, among the 38 studies, the outcome of pruritus score was researched in 18 studies. The outcome of TBA was researched in 31 studies. The outcome of ALT was researched in 34 studies. The outcome of AST was researched in 27 studies (Table 3). According to the Cochrane Reviewers’ Handbook, six studies (Wu and Yang, 2016; Liang, 2018; Liu and Yang, 2014; Luo et al., 2019; Zhu and Huang, 2008; Qiu et al., 2017) used a random number table method to group included studies. A double-blind method was used in a study (Hu and Yang, 2013).

### Risk of Bias of Included Trials

**FIGURE 2:**
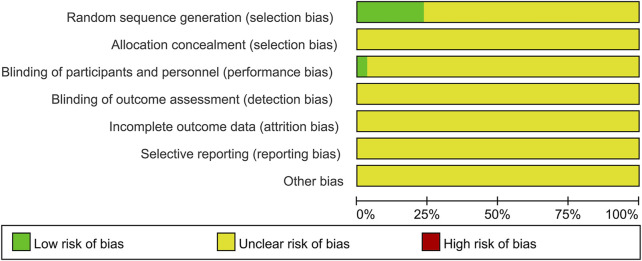
Quality assessment was performed using Review Manager 5.3 according to the Cochrane Handbook for Systematic Reviews of Interventions, version 5.2. The red square indicates a high risk of bias. The green square indicates a low risk of bias, and the blank square indicates an unclear risk of bias.

**FIGURE 3:**
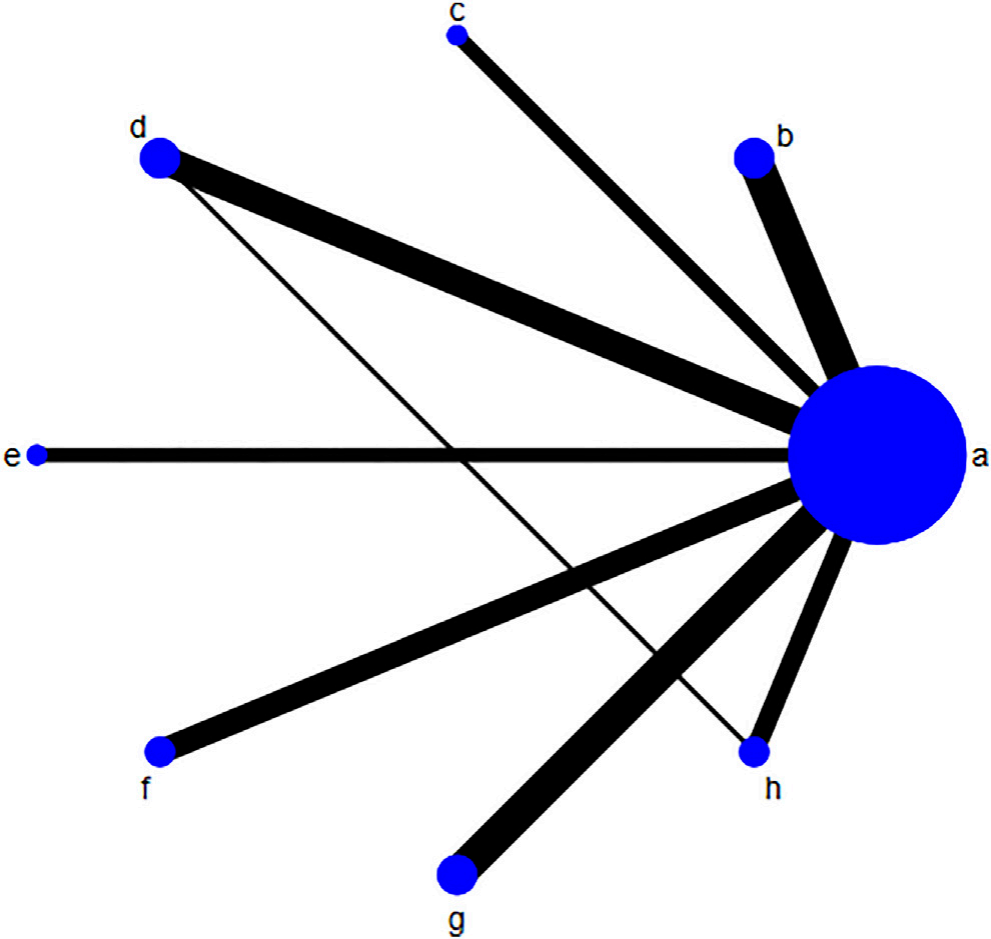
Network of comparisons for efficacy. The size of the circle shows the number of each randomized controlled trial included. The width of the lines is proportional to the comparisons of each randomized controlled trial included. Notes: **(A)** UDCA, **(B)** Yinzhihuang oral liquid + UDCA, **(C)** salvia injection + UDCA; **(D)** Yinchenhao decoction + UDCA, **(E)** the similar prescription of Yinchenhao decoction 1 + UDCA, **(F)** the similar prescription of Yinchenhao decoction 2 + UDCA, **(G)** the similar prescription of Yinchenhao decoction 3 + UDCA, and **(H)** other traditional Chinese medicine prescriptions + UDCA. UDCA, ursodeoxycholic acid.

The risk of bias for each included trial was evaluated according to the Cochrane risk of bias estimation. According to the Cochrane Reviewers’ Handbook, six studies (Wu and Yang, 2016; Liang, 2018; Liu and Yang, 2014; Luo et al., 2019; Zhu and Huang, 2008; Qiu et al., 2017) used a random number table method to group included studies. A double-blind method was used only in one study (Hu and Yang, 2013). However, other risks of quality of the included studies were not mentioned (Figure 2). Figure 3 shows the network of comparisons for efficacy. IP-a was the public control group, and other intervention plans were directly compared with IP-a (Figure 3).

### The Therapeutic Effect Ranking

**FIGURE 4:**
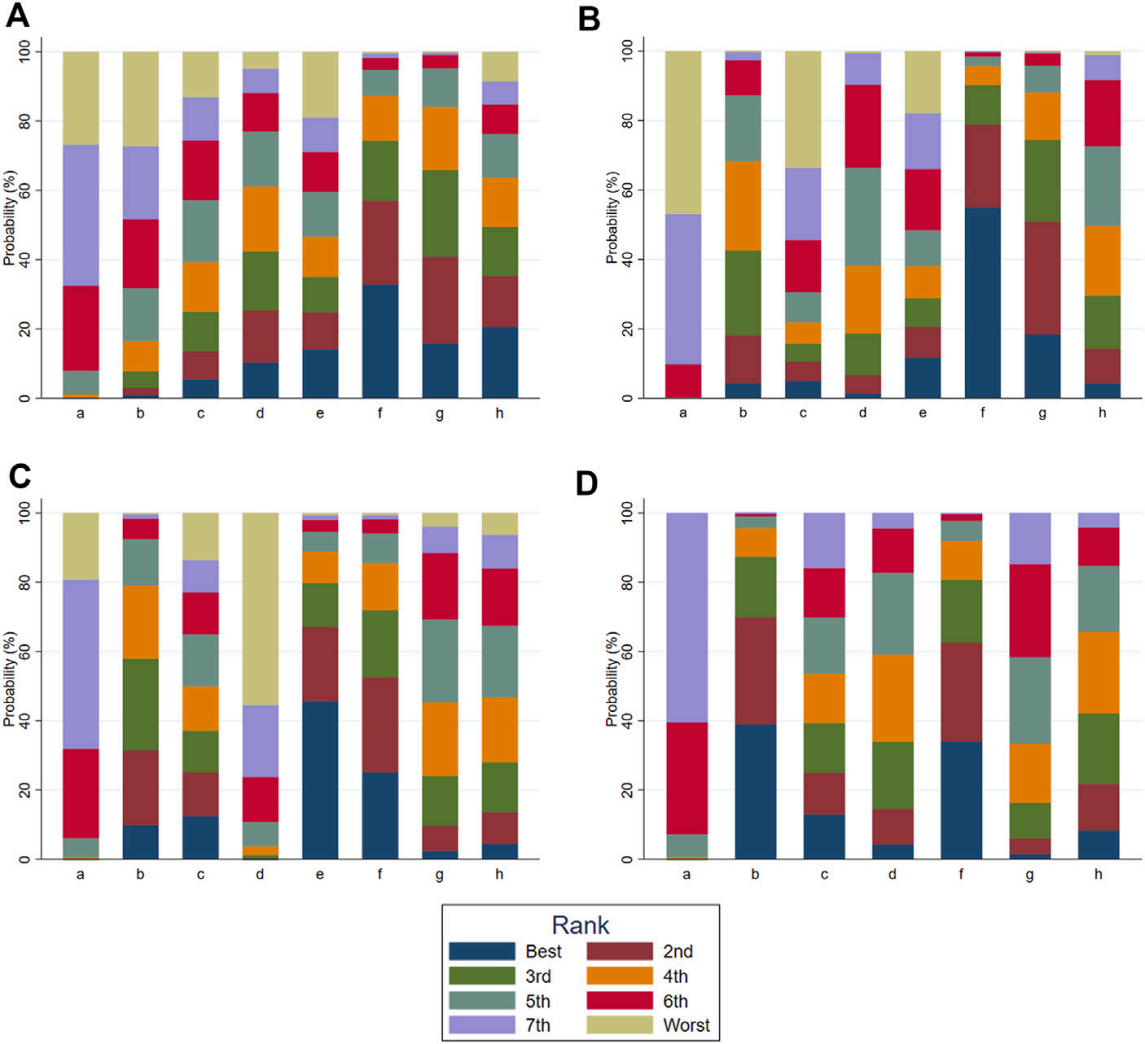
Ranking probabilities of all intervention plan at different possible rankings. Notes: a: UDCA, b: Yinzhihuang oral liquid + UDCA, c: salvia injection + UDCA, d: Yinchenhao decoction + UDCA, e: the similar prescription of Yinchenhao decoction 1 + UDCA, f: the similar prescription of Yinchenhao decoction 2 + UDCA, g: the similar prescription of Yinchenhao decoction 3 + UDCA, and h: other traditional Chinese medicine prescriptions + UDCA. **(A)** Pruritus score. **(B)** Total bile acid (TBA). **(C)** Alanine aminotransferase (ALT). **(D)** Aspartate transaminase (AST).

**FIGURE 5:**
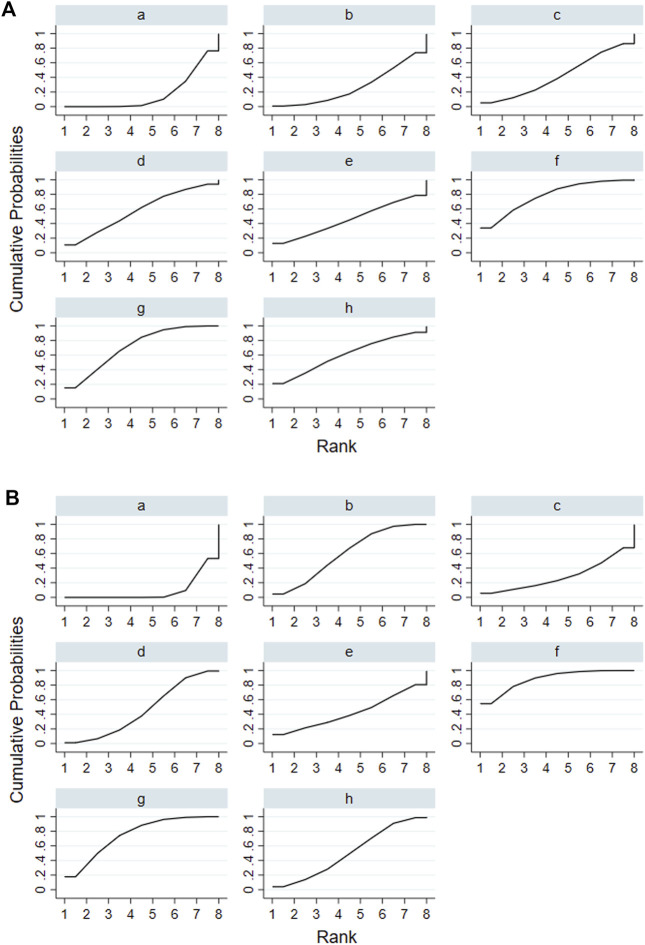
Surface under the cumulative ranking curve (SUCRA) of pruritus score and TBA. Notes: (i): UDCA, (ii): Yinzhihuang oral liquid + UDCA, (iii): salvia injection + UDCA, (iv): Yinchenhao decoction + UDCA, (v): the similar prescription of Yinchenhao decoction 1 + UDCA, (vi): the similar prescription of Yinchenhao decoction 2 + UDCA, (vii): the similar prescription of Yinchenhao decoction 3 + UDCA, and (viii): other traditional Chinese medicine prescriptions + UDCA. **(A)** Pruritus score. **(B)** TBA.

According to the tests of global inconsistency, the inconsistency model showed that all outcomes had no significant inconsistency (*p* > 0.05) (Table 4). Figure 4 shows the different probabilities when every intervention plan was at different possible rankings. For pruritus score, the probability that IP-f was the best treatment plan was the highest. The probability that IP-a was the worst treatment plan was the highest (Figure 4A). For TBA, the probability that IP-f was the best treatment plan was the highest; the probability that IP-a was the worst treatment plan was the highest (Figure 4B). For ALT, the probability that IP-e was the best treatment plan was the highest; the probability that IP-d was the worst treatment plan was the highest (Figure 4C). For AST, the probability that IP-b was the best treatment plan was the highest; the probability that IP-a was the worst treatment plan was the highest (Figure 4D). Figure 5 shows SUCRA. It shows the different cumulative probabilities when every intervention plan was at different possible rankings. Simultaneously, it also shows the trend of cumulative probability as the ranking changes. Furthermore, we got the ranking of therapeutic effect from the best to worst combining all the corresponding results. For pruritus, the therapeutic effect from the best to worst was IP-f > IP-g > IP-h > IP-d > IP-e > IP-c > IP-b > IP-a. For TBA, the therapeutic effect from the best to worst was IP-f > IP-g > IP-b > IP-h > IP-d > IP-e > IP-c > IP-a. For ALT, the therapeutic effect from the best to worst was IP-e > IP-f > IP-b > IP-h > IP-g > IP-c > IP-a > IP-d. For AST, the therapeutic effect from the best to worst was IP-b > IP-f > IP-h > IP-c > IP-d > IP-g > IP-a.

**TABLE 4:** The inconsistency of model and number of SUCRA.

Class	Primary outcomes	Inconsistency (*P*)	The number of SUCRA							
			a	b	c	d	e	f	g	h
1	Pruritus score	0.7549	16.1	28.1	41.1	58.9	46.1	77.6	72.1	60
2	TBA	0.8160	9	59.9	29	45.6	42.4	88.1	75.1	50.8
3	ALT	0.8458	17.7	67.1	48.9	11.3	82.5	75.6	48.4	48.6
4	AST	0.9236	7.2	79.6	48.2	48.2	–	79.1	34.1	53.6

Notes: a: UDCA, b: Yinzhihuang oral liquid + UDCA, c: salvia injection + UDCA; d: Yinchenhao decoction + UDCA, e: the similar prescription of Yinchenhao decoction 1 + UDCA, f: the similar prescription of Yinchenhao decoction 2 + UDCA, g: the similar prescription of Yinchenhao decoction 3 + UDCA, h: other Traditional Chinese Medicine prescriptions + UDCA.

**FIGURE 6:**
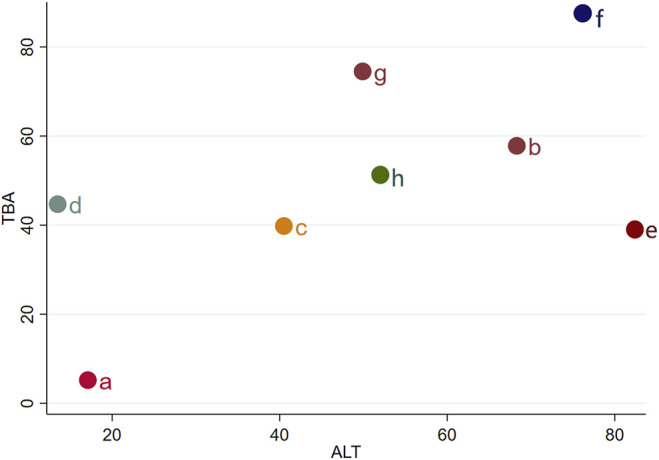
Sequence analysis of comprehensive efficacy. From the top right to bottom left, the comprehensive effect gradually decreases. Notes: a: UDCA, b: Yinzhihuang oral liquid + UDCA, c: salvia injection + UDCA, d: Yinchenhao decoction + UDCA, e: the similar prescription of Yinchenhao decoction 1 + UDCA, f: the similar prescription of Yinchenhao decoction 2 + UDCA, g: the similar prescription of Yinchenhao decoction 3 + UDCA, and h: other traditional Chinese medicine prescriptions + UDCA.

We chose TBA and ALT to analyze the comprehensive effect of each intervention plan on these two outcomes. According to the network meta-analysis, the comprehensive therapeutic effect of intervention plans could be divided into six levels. The six levels from the best to worst are as follows: the first level included IP-f; the second level included IP-g, IP-b, and IP-e; the third level included IP-h; the fourth level included IP-c; the fifth level included IP-d; and the sixth level included IP-a (Figure 6).

### Result Clarification of Network Meta-Analysis

**FIGURE 7:**
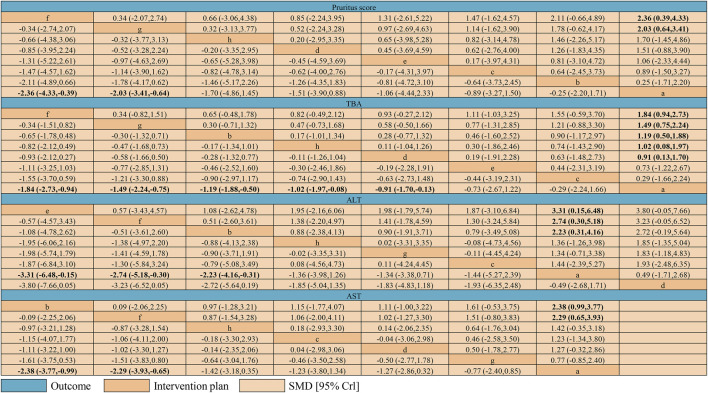
League table of network meta-analysis of pruritus score, TBA, ALT, and AST. Intervention plans are in order of each outcome ranking basing on SUCRAs. The table should be read from the top left to the bottom right. Notes: a: UDCA, b: Yinzhihuang oral liquid + UDCA, c: salvia injection + UDCA, d: Yinchenhao decoction + UDCA, e: the change of Yinchenhao decoction 1 + UDCA, f: the change of Yinchenhao decoction 2 + UDCA, g: the change of Yinchenhao decoction 3 + UDCA, and h: other traditional Chinese medicine prescriptions + UDCA.

**FIGURE 8:**
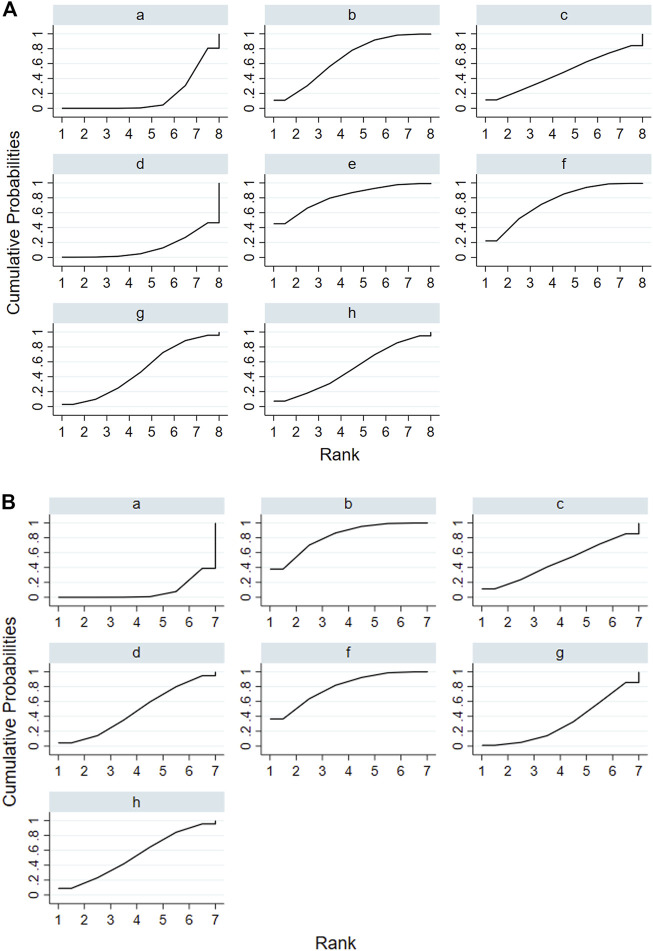
Surface under the cumulative ranking curve (SUCRA) of ALT and AST. Notes: (i): UDCA, (ii): Yinzhihuang oral liquid + UDCA, (iii): salvia injection + UDCA, (iv): Yinchenhao decoction + UDCA, (v): the similar prescription of Yinchenhao decoction 1 + UDCA, (vi): the similar prescription of Yinchenhao decoction 2 + UDCA, (vii): the similar prescription of Yinchenhao decoction 3 + UDCA, and (viii): other traditional Chinese medicine prescriptions + UDCA. **(A)** ALT. **(B)** AST.

Figure 7 shows the results of the network meta-analyses for the primary outcomes. In the outcome of pruritus score, the therapeutic effect of IP-a was significantly lower than that of IP-f [SMD −2.36, 95% credible interval (CrI) −4.33, −0.39] and IP-g (SMD −2.03, 95% CrI −3.41, −0.64) (Figure 5A). In the outcome of TBA, the therapeutic effect of IP-a was substantially lower than that of IP-f (SMD −1.84, 95% CrI −2.73, −0.94), IP-g (SMD −1.49, 95% CrI −2.24, −0.75), IP-b (SMD −1.49, 95% CrI −2.24, −0.75), IP-h (SMD −1.02, 95% CrI −1.97, −0.08), and IP-d (SMD −0.91, 95% CrI −1.70, −0.13) (Figure 5B). In the outcome of ALT, the therapeutic effect of IP-a was dramatically lower than that of IP-e (SMD −3.31, 95% CrI −6.48, −0.15), IP-f (SMD −2.74, 95% CrI −5.18, −0.30), and IP-b (SMD −2.23, 95% CrI −4.16, −0.31) (Figure 8A). In the outcome of AST, the therapeutic effect of IP-a was lower than that of IP-b (SMD −2.38, 95% CrI −3.77, −0.99) and IP-f (SMD −2.29, 95% CrI −3.93, −0.65) (Figure 8B).

## Discussion

The activation of hepatic stellate cells (HSCs) is the key factor in the liver fibrosis process. Scoparone could inhibit Smad3 phosphorylation level; ROS production; and the expression of α-smooth muscle actin (α-SMA), collagen I, and NADPH oxidase (NOX) isoforms in HSC-T6 cells. The above content showed that scoparone had potential therapeutic effects on improving liver fibrosis (Liu and Zhao, 2017). *Gardenia* is also named zhizi in China. Geniposide, quercetin, and crocin are the main active constituents of *Gardenia* (Lai et al., 2020). Among these active constituents, quercetin and crocin could increase the levels of autophagy-related protein (LC3, beclin-1, Atg5, and p-ANPK) in the liver, further reducing fat accumulation in the liver (Lai et al., 2020). For the other active constituent, geniposide had multiple pharmacological activities that were related to liver protection (Zhang C. et al., 2020; Fan et al., 2020). A research found that geniposide could inhibit STAT3/Sp1-dependent VEGF overexpression in HCC angiogenesis. The mechanism was related to the direct suppression effect of geniposide on TLR4/MyD88 activation (Zhang C. et al., 2020). *Rhubarb* is also named dahuang in China. *Rhubarb* also contains many active constituents with pharmacological activities such as rhein, emodin, and danthron (Xian et al., 2020; Zhang N. et al., 2020; Ma et al., 2021). Rhein is the main active constituent of *Rhubarb* with pharmacological activity. A research found that rhein helped maintain bile acid homeostasis in rats. The mechanism was associated with upregulating the expression of Mrp3 mRNA and FXR mRNA (Xian et al., 2020). ICP is frequently accompanied by the damage to the liver. Simultaneously, abnormal liver function indicators are the main manifestation. Therefore, the pharmacological activities of Yinchenhao, Zhizi, and Dahuang explain well why Yinchenhao decoction could improve cholestasis.

According to the result of our research, the probability that IP-f [the change of Yinchenhao decoction 2 (*A. capillaris Thunb* >15 g, *Gardenia* >9 g, and *Rhubarb* <5 g) + UDCA] was the best therapeutics was the highest in eight therapies. We found the dose of *Rhubarb* was reduced in IP-f. Combined with the pharmacological activities of *A. capillaris Thunb*, *Gardenia*, and *Rhubarb*, the main pharmacological activities of *A. capillaris Thunb* and *Gardenia* included anti-inflammatory, anti-oxidation, and anti-fibrotic (Han JM. et al., 2013; Hui et al., 2020; Shen et al., 2020). All of these play an important role in protecting the liver. Relative to *A. capillaris Thunb* and *Gardenia*, *Rhubarb* can also reduce the level of bile acid (Xian et al., 2020). At the same time, we know the level of bile acid in the human body is dynamically balanced. Therefore, we guessed using too much *Rhubarb* could break the dynamic balance. Also, the level of bile acid is harmful whether it is too high or too low. This can be seen as an explanation why the dose of *Rhubarb* is reduced in IP-f. The probability that IP-a (UDCA only) was the worst therapeutics was the highest in eight therapies. At the same time, the result showed the intervention plans of combination medication are generally better than UDCA alone in terms of improving pruritus and the level of TBA, ALT, and AST. It was only an accident that IP-a was better than IP-d in terms of improving the level of ALT. The research process is as follows, at first, we calculated the probability of different intervention plans at different possible rankings. In terms of improving pruritus and the level of TBA and AST, the probability that IP-f is the best therapeutics is the highest. At the same time, in terms of improving the level of ALT, although the probability that IP-f was the best therapeutics was not the highest, we found the probability that IP-f was the second-ranked therapeutics was the highest. Not only that, IP-f still had the highest probability of being the best therapy compared with other therapies except IP-e. In addition, the result showed that the probability that IP-a was the worst therapeutics was the highest in terms of improving pruritus and the level of TBA and AST. In terms of improving the level of ALT, the probability that only IP-d was the worst therapy was higher than that of IP-a. Furthermore, we chose the outcomes of ALT and TBA to conduct a comprehensive analysis. According to the result, the comprehensive therapeutic effect of intervention plans was divided into six levels by our group. The six levels from the best to worst are as follows: the first level included IP-f; the second level included IP-g, IP-b, and IP-e; the third level included IP-h; the fourth level included IP-c; the fifth level included IP-d; and the sixth level included IP-a. The result showed that IP-f still is the best therapeutic. Finally, the league table was used to show the comparison result between two intervention plans. The result showed that the quality of evidence was low in most comparisons. In the outcome of pruritus score, the therapeutic effect of IP-a was significantly lower than that of IP-f (SMD −2.36, 95% CrI −4.33, −0.39) and IP-g (SMD −2.03, 95% CrI −3.41, −0.64). In the outcome of TBA, the therapeutic effect of IP-a was significantly lower than that of IP-f (SMD −1.84, 95% CrI −2.73, −0.94), IP-g (SMD −1.49, 95% CrI −2.24, −0.75), IP-b (SMD −1.49, 95% CrI −2.24, −0.75), IP-h (SMD −1.02, 95% CrI −1.97, −0.08), and IP-d (SMD −0.91, 95% CrI −1.70, −0.13). In the outcome of ALT, the therapeutic effect of IP-a was significantly lower than that of IP-e (SMD −3.31, 95% CrI −6.48, −0.15), IP-f (SMD −2.74, 95% CrI −5.18, −0.30), and IP-b (SMD −2.23, 95% CrI −4.16, −0.31). In the outcome of AST, the therapeutic effect of IP-a was lower than that of IP-b (SMD −2.38, 95% CrI −3.77, −0.99) and IP-f (SMD −2.29, 95% CrI −3.93, −0.65).

This study used a novel method to divide Chinese prescriptions into different groups. The method contributes to systematic research for Chinese prescriptions. This study systematically researched and compared different Chinese prescriptions and patent medicine combine with UDCA in the treatment efficacy for intrahepatic cholestasis of pregnancy. In this study, the result showed the probability of every intervention plan at different possible rankings. Simultaneously, the result also showed the comparison between different intervention plans. More than that, an interesting result was found. We found the similar prescription of Yinchenhao decoction 2 (*A. capillaris Thunb* >15 g, *Gardenia* >9 g, and *Rhubarb* <5 g) has the best synergy with ursodeoxycholic acid. The dosage of the main component in Yinchenhao decoction 2 is different from traditional Yinchenhao decoction. This finding broke up the traditional Yinchenhao decoction’s dosage that has been used for thousands of years. At the same time, a clear dosage range of the main component gave a basis for the modern clinical use of Yinchenhao decoction. However, this study does have some limitations. First, the small sample sizes of included trials in this study were not sufficient to evaluate suitable therapeutic drugs for the treatment of ICP in clinical practice. Second, with different clinical characteristics in the studies, unbalanced baselines still existed. Third, the quality of our analysis is limited by the quality of the underlying data. In the *Risk of Bias of Included Trials* section, the allocation concealment (selection bias), blinding of outcome assessment (detection bias), incomplete outcome data (attrition bias), and selective reporting (reporting bias) were not reported in all trials. Furthermore, most of the study groups included in this study were indirect comparisons, and there was a lack of direct comparisons among different combination therapies. Since the consistency model of high evidence quality could not be fitted during analysis, the reliability of the resulting data could only be used as a reference. As a consequence of these limitations, we clearly find that the overall level of clinical research needs to be improved. Therefore, more rigorous and well-designed RCTs are needed to confirm these findings.

## Conclusion

According to the result of our research, the intervention plans of combination medication were generally better than UDCA alone in terms of improving pruritus and the level of TBA, ALT, and AST. However, IP-a was better than IP-d in terms of improving the level of ALT. Furthermore, the probability that IP-f [the similar prescription of Yinchenhao decoction 2 (*A. capillaris Thunb* >15 g, *Gardenia* >9 g, and *Rhubarb* <5 g) + UDCA] was the best therapeutics was the highest in eight therapies. In addition, according to the result, this study indicated that the proportion of medicine used is different from the traditional proportion of Yinchenhao decoction. Therefore, the dose of IP-f might be a beneficial therapeutic method for the clinical medication of ICP.

## Data Availability

The original contributions presented in the study are included in the article/Supplementary Material; further inquiries can be directed to the corresponding authors.

## Abbreviations

ALT, alanine aminotransferase; AST, aspartate transaminase; CBM, China Biology Medicine; CNKI, Chinese databases including China National Knowledge Infrastructure; HSCs, hepatic stellate cells; ICP, intrahepatic cholestasis of pregnancy; IP-a, intervention plan a; IP-b, intervention plan b; NMA, network meta-analysis; TBA, total bile acid; RCT, randomized controlled trial; SMD, standardized mean difference; SUCRA, surface under the cumulative ranking curve; UDCA, ursodeoxycholic acid; α-SMA, α-smooth muscle actin.
