# Supplementary material for: Comparative Evidence for Intrahepatic Cholestasis of Pregnancy Treatment With Traditional Chinese Medicine Therapy: A Network Meta-Analysis
Source: Front Pharmacol. 2021 Nov 30;12:774884. doi: 10.3389/fphar.2021.774884 (PMC8670235; doi:10.3389/fphar.2021.774884)
Supplement: Supplementary file 1 [file Table1.DOCX]

**EXCESS TABLE 1 |** Formulation, main botanical, concentration or source of the included studies.

|  | Study | Formulation, main botanical, concentration or source | | | | | Quality control  (Y/N) | | Chemical analysis (Y/N) |
| --- | --- | --- | --- | --- | --- | --- | --- | --- | --- |
| 1 | Jiang 2014(Jiang, 2014) | Yinzhihuang oral liquid | | Beijing China Resources High-tech Natural Medicine Co., Ltd. | Artemisia scoparia Waldst. & Kit. [Asteraceae; Artemisia scoparia extract]  Gardenia jasminoides J. Ellis [Rubiaceae; Gardenia jasminoides extract]  Scutellaria baicalensis Georgi [Lamiaceae; Scutellaria baicalensis extract]  Lonicera japonica Thunb. [Caprifoliaceae; Lonicera japonica extract] | | Y-According to first part of *“Pharmacopoeia of The People's Republic of China”* on page 1235 and 1236 | | Y- HPLC |
| 2 | Yuan 2014(Yuan & Lan, 2014) |  |  | Beijing Shuanghe High-tech Natural Medicine Co., Ltd. |  |  |  |  |  |
| 3 | Deng 2015(Deng, 2015) |  |  | — |  |  |  |  |  |
| 4 | Dai 2016(Dai, 2016) |  |  | Beijing China Resources High-tech Natural Medicine Co., Ltd. |  |  |  |  |  |
| 5 | Wu 2016(M. Wu & Yang, 2016) |  |  | Beijing China Resources High-Tech Natural Medicine Co., Ltd. |  |  |  |  |  |
| 6 | Bo 2017(Bo, 2017) |  |  | — |  |  |  |  |  |
| 7 | Zhang 2017(Y. H. Zhang, 2017) |  |  | Beijing China Resources High-Tech Natural Medicine Co., Ltd. |  |  |  |  |  |
| 8 | Liang 2018(Liang, 2018) |  |  | Beijing Shuanghe High-tech Natural Medicine Co., Ltd. |  |  |  |  |  |
| 9 | Yu 2018(Yu & Chen, 2018) |  |  | Beijing China Resources High-Tech Natural Medicine Co., Ltd. |  |  |  |  |  |
| 10 | Fang 2009(J. Fang et al., 2009) | Salvia injection | | Zhengda Qingchunbao Pharmaceutical Co., Ltd. | | | Y-According to the first part of *“Pharmacopoeia of The People's Republic of China”* on page 77 and 78 | | Y-HPLC |
| 11 | Pan 2013(Pan et al., 2013) |  |  | Salvia miltiorrhiza Bunge [Lamiaceae; Salviae miltiorrhizae radix et rhizoma] 0.4g | | |  |  |  |
| 12 | Peng 2015(Peng, 2015) |  |  | Salvia miltiorrhiza Bunge [Lamiaceae; Salviae miltiorrhizae radix et rhizoma] | | |  |  |  |
| 13 | Hua 2010(Hua, 2010) | Yinchenhao decoction | Artemisia scoparia Waldst. & Kit. [Asteraceae; Artemisia scoparia dried aerial part]. | | | Prepared by Hua 2010(Hua, 2010) | | Y-Prepared basing on *“Treatise on Febrile Diseases”*  *Artemisia scoparia* comply with the first part of *“Pharmacopoeia of The People's Republic of China”* on page 250 and 251  *Gardenia jasminoides* comply with the first part of *“Pharmacopoeia of The People's Republic of China”* on page 259 and 260  *Rheum palmatum* comply with the first part of *“Pharmacopoeia of The People's Republic of China”* on page 24 and 25 | Y-HPLC |
|  |  |  | Gardenia jasminoides J.Ellis [Rubiaceae; Gardenia jasminoides dried ripe fruit].  Rheum palmatum L. [Polygonaceae; Rheum palmatum dried radix et rhizoma]. | | |  |  |  |  |
| 14 | Wu 2010(Z. Y. Wu, 2010) |  | Artemisia scoparia Waldst. & Kit. [Asteraceae; Artemisia scoparia dried aerial part] 30g | | | Prepared by Wu 2010(Z. Y. Wu, 2010) | |  |  |
|  |  |  | Gardenia jasminoides J.Ellis [Rubiaceae; Gardenia jasminoides dried ripe fruit] 10g | | |  |  |  |  |
| 15 | Liu 2014(P. Z. Liu & Yang, 2014) |  | Artemisia scoparia Waldst. & Kit. [Asteraceae; Artemisia scoparia dried aerial part] 30g | | | Prepared by Liu 2014(P. Z. Liu & Yang, 2014) | |  |  |
|  |  |  | Rheum palmatum L. [Polygonaceae; Rheum palmatum dried radix et rhizoma] 6g | | |  |  |  |  |
| 16 | Fu 2017(Fu, 2017) |  | Gardenia jasminoides J.Ellis [Rubiaceae; Gardenia jasminoides dried ripe fruit] 10g | | | Prepared by Fu 2017(Fu, 2017) | |  |  |
|  |  |  | Rheum palmatum L. [Polygonaceae; Rheum palmatum dried radix et rhizoma] 6g | | |  |  |  |  |
| 17 | Luo 2019(Z. P. Luo et al., 2019) |  | Artemisia scoparia Waldst. & Kit. [Asteraceae; Artemisia scoparia dried aerial part] 24g | | | Prepared by Luo 2019(Z. P. Luo et al., 2019) | |  |  |
|  |  |  | Gardenia jasminoides J.Ellis [Rubiaceae; Gardenia jasminoides dried ripe fruit] 12g | | |  |  |  |  |
| 18 | Zhu 2008(Zhu & Huang, 2008) |  | Artemisia scoparia Waldst. & Kit. [Asteraceae; Artemisia scoparia dried aerial part] 15g | | | Prepared by Zhu 2008(Zhu & Huang, 2008) | |  |  |
|  |  |  | Rheum palmatum L. [Polygonaceae; Rheum palmatum dried radix et rhizoma] 6g | | |  |  |  |  |
| 19 | Wang 2011A(J. D. Wang & Lai, 2011) |  | Artemisia scoparia Waldst. & Kit. [Asteraceae; Artemisia scoparia dried aerial part] 25g | | | Prepared by Wang 2011A(J. D. Wang & Lai, 2011) | |  |  |
|  |  |  | Gardenia jasminoides J.Ellis [Rubiaceae; Gardenia jasminoides dried ripe fruit] 8g | | |  |  |  |  |
| 20 | Wang 2016(X. Wang et al., 2016) |  | Artemisia scoparia Waldst. & Kit. [Asteraceae; Artemisia scoparia dried aerial part] 18g | | | Prepared by Wang 2016(X. Wang et al., 2016) | |  |  |
|  |  |  | Gardenia jasminoides J.Ellis [Rubiaceae; Gardenia jasminoides dried ripe fruit] 8g | | |  |  |  |  |
| 21 | Wang 2007(Y. P. Wang & Hu, 2007) |  | Artemisia scoparia Waldst. & Kit. [Asteraceae; Artemisia scoparia dried aerial part] 20g | | | Prepared by Wang 2007(Y. P. Wang & Hu, 2007) | |  |  |
|  |  |  | Rheum palmatum L. [Polygonaceae; Rheum palmatum dried radix et rhizoma] 6g | | |  |  |  |  |
| 22 | Xie 2015(Xie et al., 2015) |  | Artemisia scoparia Waldst. & Kit. [Asteraceae; Artemisia scoparia dried aerial part] | | | Prepared by Xie 2015(Xie et al., 2015) | |  |  |
|  |  |  | Gardenia jasminoides J.Ellis [Rubiaceae; Gardenia jasminoides dried ripe fruit] 10g | | |  |  |  |  |
| 23 | Qiu 2017(Qiu et al., 2017) |  | Artemisia scoparia Waldst. & Kit. [Asteraceae; Artemisia scoparia dried aerial part] 30g . | | | Prepared by Qiu 2017(Qiu et al., 2017) | |  |  |
|  |  |  | Gardenia jasminoides J.Ellis [Rubiaceae; Gardenia jasminoides dried ripe fruit] | | |  |  |  |  |
| 24 | Fang 2018(F. M. Fang et al., 2018) |  | Artemisia scoparia Waldst. & Kit. [Asteraceae; Artemisia scoparia dried aerial part] 15g | | | Prepared by Fang 2018(F. M. Fang et al., 2018) | |  |  |
|  |  |  | Gardenia jasminoides J.Ellis [Rubiaceae; Gardenia jasminoides dried ripe fruit] 12g | | |  |  |  |  |
| 25 | Guo 2018(Guo & Pei, 2018) |  | Artemisia scoparia Waldst. & Kit. [Asteraceae; Artemisia scoparia dried aerial part] 15g | | | Prepared by Guo 2018(Guo & Pei, 2018) | |  |  |
|  |  |  | Gardenia jasminoides J.Ellis [Rubiaceae; Gardenia jasminoides dried ripe fruit] 9g | | |  |  |  |  |
| 26 | Wang 2009(X. Wang, 2009) |  | Artemisia scoparia Waldst. & Kit. [Asteraceae; Artemisia scoparia dried aerial part] 15g | | | Prepared by Wang 2009(X. Wang, 2009) | |  |  |
|  |  |  | Gardenia jasminoides J.Ellis [Rubiaceae; Gardenia jasminoides dried ripe fruit] 6g | | |  |  |  |  |
| 27 | Wang 2011B(J. J. Wang & Du, 2011) |  | Artemisia scoparia Waldst. & Kit. [Asteraceae; Artemisia scoparia dried aerial part] 15g | | | Prepared by Wang 2011B(J. J. Wang & Du, 2011) | |  |  |
|  |  |  | Gardenia jasminoides J.Ellis [Rubiaceae; Gardenia jasminoides dried ripe fruit] 5g | | |  |  |  |  |
| 28 | Lv 2013(Lv, 2013) |  | Artemisia scoparia Waldst. & Kit. [Asteraceae; Artemisia scoparia dried aerial part] 30g | | | Prepared by Lv 2013(Lv, 2013) | |  |  |
| 29 | Han 2013(X. Han et al., 2013) |  | Artemisia scoparia Waldst. & Kit. [Asteraceae; Artemisia scoparia dried aerial part] 15g | | | Prepared by Han 2013(X. Han et al., 2013) | |  |  |
| 30 | Chen 2014(Chen et al., 2014) |  | Artemisia scoparia Waldst. & Kit. [Asteraceae; Artemisia scoparia dried aerial part] 15g | | | Prepared by Chen 2014(Chen et al., 2014) | |  |  |
| 31 | Yin 2015(Yin & Zhou, 2015) |  | Artemisia scoparia Waldst. & Kit. [Asteraceae; Artemisia scoparia dried aerial part] 15g | | | Prepared by Yin 2015(Yin & Zhou, 2015) | |  |  |
|  |  |  | Gardenia jasminoides J.Ellis [Rubiaceae; Gardenia jasminoides dried ripe fruit] 3g | | |  |  |  |  |
| 32 | Gao 2017(W. J. Gao & Zhao, 2017) |  | Artemisia scoparia Waldst. & Kit. [Asteraceae; Artemisia scoparia dried aerial part] 15g | | | Prepared by Gao 2017(W. J. Gao & Zhao, 2017) | |  |  |
| 33 | Luo 2017(L. Luo & Chen, 2017) |  | Artemisia scoparia Waldst. & Kit. [Asteraceae; Artemisia scoparia dried aerial part] 15g | | | Prepared by Luo 2017(L. Luo & Chen, 2017) | |  |  |
| 34 | Hu 2013(Hu & Yang, 2013) |  | Artemisia scoparia Waldst. & Kit. [Asteraceae; Artemisia scoparia dried aerial part] 10g | | | Prepared by Hu 2013(Hu & Yang, 2013) | |  |  |
| 35 | He 2014(He et al., 2014) |  | Artemisia scoparia Waldst. & Kit. [Asteraceae; Artemisia scoparia dried aerial part] 12g | | | Prepared by He 2014(He et al., 2014) | |  |  |
| 36 | Wei 2016(Wei, 2016) |  | Artemisia scoparia Waldst. & Kit. [Asteraceae; Artemisia scoparia dried aerial part] 10g | | | Prepared by Wei 2016(Wei, 2016) | |  |  |
|  |  |  | Gardenia jasminoides J.Ellis [Rubiaceae; Gardenia jasminoides dried ripe fruit] 10g | | |  |  |  |  |
| 37 | Nie 2019(Nie & Lu, 2019) |  | Artemisia scoparia Waldst. & Kit. [Asteraceae; Artemisia scoparia dried aerial part] 10g | | | Prepared by Nie 2019(Nie & Lu, 2019) | |  |  |
|  |  |  | Rheum palmatum L. [Polygonaceae; Rheum palmatum dried radix et rhizoma] 3g | | |  |  |  |  |
| 38 | Gu 2014(Gu et al., 2014) |  | Artemisia scoparia Waldst. & Kit. [Asteraceae; Artemisia scoparia dried aerial part] 20g or 12g | | | Prepared by Gu 2014(Gu et al., 2014) | |  |  |
|  |  |  | Gardenia jasminoides J.Ellis [Rubiaceae; Gardenia jasminoides dried ripe fruit] 10g | | |  |  |  |  |
|  |  |  | Rheum palmatum L. [Polygonaceae; Rheum palmatum dried radix et rhizoma] 3g | | |  |  |  |  |

Notes: Y, reported; N, not reported.
